# Epigenetic pathways in PTSD

**DOI:** 10.1192/j.eurpsy.2021.120

**Published:** 2021-08-13

**Authors:** T. Klengel

**Affiliations:** Department Of Psychiatry, McLean Hospital and University Medical Center Goettingen, Belmont, United States of America

## Abstract

**Abstract Body:**

Nonhuman primates (NHPs) are critical for translational research due to their close genetic, physiological, and behavioral similarity to humans. In particular, higher brain functions depend on brain regions and neural circuits that evolved differently between primates and rodents. Thus, NHPs are a strong translational model system to investigate the pathophysiology and relevant biological correlates of mental disorders. This talk will focus on translational approaches leveraging NHP models to advance our understanding of environmentally induced epigenetic changes in post-traumatic stress disorder (PTSD). Environmental factors including early life stress significantly contribute to risk and resilience for psychiatric disorders including PTSD. However, human studies are often confounded, and it remains challenging to identify robust epigenetic signals in clinical populations even in large studies. We investigate the natural spectrum of behavioral phenotypes in rhesus macaques to complement human studies with a focus on stress and fear. This talk will present data on epigenetic signatures of fear and the effects of early life stress in rhesus monkeys and their relationship to human studies.

**Disclosure:**

No significant relationships.

